# Effect of Lactic Acid Bacteria and Propionic Acid on Fermentation Characteristics, Chemical Composition, and Aerobic Stability of High-Moisture Corn Grain Silage

**DOI:** 10.3390/microorganisms13010033

**Published:** 2024-12-27

**Authors:** Jinze Bao, Lei Wang, Zhu Yu

**Affiliations:** College of Grassland Science and Technology, China Agricultural University, Beijing 100193, China; bs20203240986@cau.edu.cn (J.B.); wanglei0622@cau.edu.cn (L.W.)

**Keywords:** aerobic stability, high-moisture corn, fermentation characteristics

## Abstract

This investigation aimed to assess the effect of additives on the aerobic stability, fermentation profile, and chemical composition of high-moisture corn grain silage. The corn grain was milled and divided this into four distinct treatment groups: *Lentilactobacillus buchneri*, propionic acid, *Lactiplantibacillus plantarum*, and no additive (control). The capacity of the silos was 1 L and density was 1000 kg/m^3^. Each group had three replicates and was fermented for 45 d. At silo opening, one part of silage was used for fermentation parameters, chemical composition, and in vitro dry matter digestibility analysis; another part was used for aerobic stability determination. Compared with the control, all additives increased lactic acid and dry matter concentrations (*p* < 0.001) and decreased neutral detergent fiber level (*p* < 0.001). In comparison with the control, the application of *Lentilactobacillus buchneri* and propionic acid improved silage aerobic stability, showed by lower pH level and yeast and mold populations after exposure to air. The findings offer theoretical groundwork and technological backing for the use of high-moisture corn grain silage.

## 1. Introduction

High-moisture corn grain silage (HMCS) refers to the corn kernel physiological maturity, moisture content of 28% to 32% when threshing directly after crushing, compaction and sealing fermentation, proliferation of lactic acid bacteria, modulation for cattle, sheep, swine, and chickens and other livestock and poultry feed, with a long preservation time, high nutritive value, good feeding effect, and so on. Compared with harvesting traditional kernel corn, HMCS has the following advantages: 1. Kernel harvesting time is 2 to 3 weeks earlier than harvesting dry kernels, which can effectively reduce the impact of unfavorable weather and other factors and alleviate the pressure of labor and mechanical equipment during harvesting. 2. The moisture content of freshly harvested corn is higher, and the safe storage moisture requirement of corn is under 14%, and the airing and drying process of corn not only generates a lot of cost. It also greatly increases the risk of corn contamination by mycotoxins. For more than 70 years, high-moisture corn has been extensively utilized in North America and Europe as a premium livestock feed [1,2]. HMCS during the feeding phase is a frequent issue in dairy farms. The high-moisture corn at this stage has warm spots where mold can grow, which raises the possibility of mycotoxin contamination [3], affecting profits [4]. It is important to limit the metabolic pathways, such as the *clostridia*-mediated butyric acid fermentation and the production of ethanol by yeast to preserve the highest quality aspects of silage [5]. Moreover, yeast and mold may result in a waste of approximately 20% stockpiled dry matter (DM) content. They are also responsible for reducing the nutritional values at the aerobic phase, once the silo is opened. The total mixed rations and silage deterioration further lead to loss of economy along with DM loss, such as lower feed intake or deleterious impact on animal health because of the mycotoxin production [6,7,8].

Silage additives play a critical role in reducing the negative impact that undesirable silage microorganisms have on aerobic stability, nutritional value, and fermentation [9,10]. Lactic acid bacteria (LAB) that are homo-fermentative can improve grass silage fermentation by increasing the recovery of DM or minimizing its loss [11]. Oliveira et al. [11] found that *Lactiplantibacillus plantarum* (LP) inoculation had no influence on aerobic stability for a variety of silage types. However, other studies have demonstrated that LP may commonly reduce aerobic stability [12,13]. There are some findings related to LP that show positive, albeit highly varied effects on the digestion of grass silage [14,15,16]. Meta-analysis from Oliveira et al. [11] revealed increased milk production with LP inoculation without affecting the digestive capabilities or DM consumption of grass and legumes prepared silage.

Obligatory hetero-fermentative LAB facilitates silage aerobic stability improvement by generating antifungal acetic acid [12,17]. *Lentilactobacillus buchneri* (LB) is the most commonly employed species in commercial additive preparations. So far, no research has been performed specifically on HMCS, and there is generally very little experimental evidence available regarding aeration’s influences on nutritional values alteration.

Thus, this study aimed to assess how various additions affected the aerobic stability, chemical composition, and fermentation profile of HMCS. The results of this study will offer new alternatives of nutritional formulation for pastures and will help to develop HMCS production in China.

## 2. Materials and Methods

In Baoding City, Hebei province, China, at the National Modern Agricultural Industrial Technology System Hebei Experimental Station (38°10′~38°40′ N, 113°40′~116°20′ E), corn was sown. Four different blocks spread over two fields were selected and harvested utilizing a randomized complete block approach. The corn was processed through a hammer mill to 5 mm after it was hand husked. After that, the corn grains were treated as follows: (1) sterilized distilled water (CON) (4 mL/kg); (2) *Lentilactobacillus buchneri* test group (LB) (1 × 10^6^ cfu /g); (3) propionic acid group (PA) (0.4 g/kg fresh weight, FW); and (4) *Lactiplantibacillus plantarum* group (LP) (1 × 10^6^ cfu/g). All treatment components were then solubilized in sterilized distilled water and sprayed evenly. Water was administered in the control group. Ground corn was put into polyethylene silos with a height of 190 mm and a diameter of 95 mm. All of the treatments were repeated thrice, and the silos had a density of 1000 kg/m^3^.

The corn silage samples were taken out of the silos after 45 days of ensiling. The sample (25 g) was combined with distilled water (225 mL) that had been sterilized, and the mixture was then turned for 2 min in the mixer. After that, four layers of filter paper (non-woven fabric) were utilized to filter the liquid. The pH of the filtrate was measured, followed by 5 min centrifuging at 4 °C and 10,000× *g*. Ammonia nitrogen (NH_3_-N) concentration was measured utilizing the phenol-hypochlorite technique [18]. Moreover, a 0.22 μm filter was utilized to pass supernatant through it, and the amount of lactic, acetic, butyric, and propionic acids was measured by high-performance liquid chromatography. The system was composed of the mobile phase of perchloric acid (3 mmol/L), an SPD-M10AVP detector, and a 30 mm × 8 mm gel column (Shodex Rspak KC-811S-DVB) that was kept at 50 °C. The injection volume was 5 µL. Detections were carried out at 210 nm [19].

Samples of raw materials and silage were placed in a forced-air oven for 48 h drying at 60 °C. Once dried, a lab grinder was utilized to grind the material and pass through a 1 mm sieve. Acid detergent fiber (ADF) and neutral detergent fiber (aNDF) were measured by an Ankom2000 fiber analyzer according to Vansoest et al. [20]. The total nitrogen content is determined by the Kjeldahl method [21] and then multiplied by a coefficient of 6.25 to obtain the crude protein (CP) content. The amount of water-soluble carbohydrate (WSC) was calculated utilizing the technique suggested by Ke et al. [22]. The AOAC technique (AOAC, 2000) was utilized to determine the ash and ether extract (EE). Utilizing the approach by McCready et al. [23], starch was calculated. In vitro dry matter digestibility (IVDMD) was performed using the Ankom RFS bottles and the pressure sensor technology described by Yuan et al. [24]

The silage’s aerobic stability was assessed at room temperature (20.6 ± 0.2 °C). The data recorders (Tinytag Talk 2, Gemini, Chichester, UK) were positioned in a plastic container’s geometric center, to which the silage was added loosely. Every 2 h, the temperatures of the silage and the chamber were measured. For 336 h, all the plastic containers were kept in separate insulated polystyrene boxes with open air. The aerobic stability was estimated by the time (h) taken for raising the silage mass’s temperature by 2 °C higher than the room temperature.

The modified Reich and Kung [25] method was utilized to count the mold, yeast, and LAB in the HMCS. To put it briefly, 10 g of material was homogenized for 1 min utilizing autoclaved normal saline (100 mL of 0.85% NaCl). Next, autoclaved normal saline was utilized to perform 10-fold serial dilutions. After cultivating the samples on MRS agar at 37 °C for 48–72 h, the number of LAB was recorded. Following incubation with the same time length at 30 °C, yeast and mold were counted on spread plates of potato dextrose agar treated with chloramphenicol. The enumerations were conducted on a suitably diluted plate containing 30–300 colonies, and microbial enumeration was recorded as log_10_ cfu/g of fresh fodder.

SPSS version 25.0 was utilized for statistical analysis. With SPSS version 25.0, two-way analyses of variance (ANOVAs) were carried out to assess the data under the fixed effects of additives and hybrid types. When interaction effects were substantial, SPSS’s GLM statement was utilized to do a straightforward effect analysis. When analyzing data with single fixed effects, one-way ANOVA or *t*-tests were utilized, and the Tukey method was utilized to compare means. Statistically significant deviations from the means were identified utilizing a significance threshold of *p* < 0.05.

## 3. Results

### 3.1. Chemical Composition of Raw Materials

The non-fermented high-moisture corn’s chemical composition is shown in Table 1.

### 3.2. Fermentation Profiles of Corn Grain Silage

Table 2 displays the variations in pH and organic acid contents in HMCS for 45-day ensiling. The pH value of HMCS was less than 3.9 after 45 years of silage fermentation, and it had no discernible impact on any of the treatments (*p* < 0.05). The addition of LB and LP test groups was substantially greater than that of the PA test group (*p* < 0.05); the three additives raised the LA content of HMCS substantially (*p* < 0.05), in comparison with the control. The AA content of HMCS can be greatly increased by adding LB and PA (*p* < 0.05); the LB test group had a substantially greater AA content than the PA test group (*p* < 0.05), and adding LP has no substantial effect (*p* < 0.05). The addition of the PA test group was substantially higher than those of the other three test groups in terms of propionic acid concentration (*p* < 0.05). The NH_3_-N of HMCS was unaffected by any of the treatments (*p* < 0.05).

### 3.3. Chemical Composition of HMCS

Table 3 displays the chemical makeup, dry matter, and in vitro dry matter digestibility of HMCS with various ensiling additives treatment. The DM content of HMCS was considerably influenced by the chemical treatment compared with the control (*p* < 0.05). The DM content of HMCS was substantially enhanced by all three additives (*p* < 0.05). The ADF content of HMCS was substantially decreased by adding LB and LP (*p* < 0.05), but it was not substantially affected by adding PA (*p* < 0.05). The contents of starch, CP, EE, and in vitro dry matter digestibility of HMCS were not substantially influenced by any treatment (*p* < 0.05). The aNDF content in HMCS was substantially decreased in the three additive test groups, while those in LB and LP test groups were substantially lower in comparison to the PA test groups (*p* < 0.05).

### 3.4. Impact of Additives on Aerobic Stability of Corn Grain Silage

**Figure 1:**
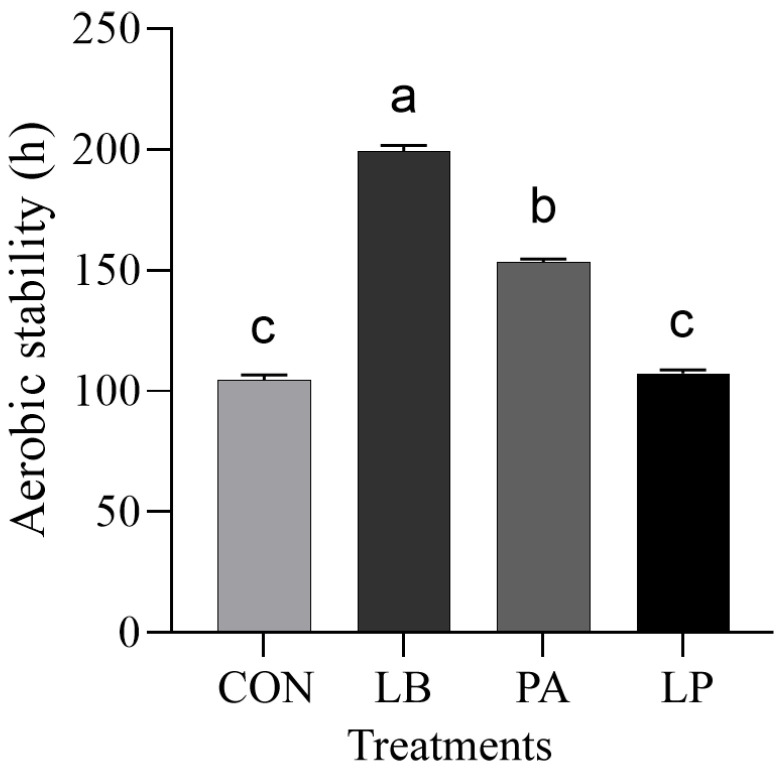
Aerobic stability of HMCS. Treatment: CON, control, no additive; LB, *Lentilactobacillus buchner*; PA, Propionic acid; LP, *Lactiplantibacillus plantarum*. Bars with different lowercase letters differed at *p* < 0.05.

The aerobic stability of HMCS when exposed to the air is displayed in Figure 1. LB and PA addition can substantially increase (*p* < 0.05) the aerobic stability of HMCS, compared with the control group. The LB group has the highest aerobic stability (206 h, followed by the PA group (156 h), while the LP group has no discernible impact (*p* > 0.05).

Table 4 displays changes in HMCS fermentative properties following exposure (aerobic). Despite the stable pH values remained in the LB and PA groups, those of all test groups were substantially elevated following about 7 d aerobic exposure (*p* < 0.05). All test groups’ pH was relatively stable for 5 days prior to exposure (aerobic) and started to rise substantially to more than 4.5 on d 5–7 of exposure (aerobic) (*p* < 0.05). Compared with the PA and LB groups, the control and LP groups were much higher. Following 5 d exposure (aerobic), no substantial change was found in LA content between the two groups, i.e., control and LP groups (*p* > 0.05). However, following 7 d exposure (aerobic), there was a substantial drop in LA contents within the two groups (*p* < 0.05). Throughout the whole 7 d exposure (aerobic) period, there was no discernible LA content difference between the PA and LB groups (*p* > 0.05). The AA content of all the test groups was consistent, 5 d prior to exposure (aerobic) and dramatically dropped 5–7 d after (*p* < 0.05). Throughout the entire 7 d exposure, the test groups did not show discernible changes in the PA content (*p* > 0.05). Five days before to exposure (aerobic), the NH_3_-N content of every test group was constant; nevertheless, it substantially increased (*p* < 0.05) between d 5 and 7 after exposure (aerobic). The NH_3_-N concentrations in the control and LP groups, following 7 d exposure (aerobic) was 12.18% and 13.31%, respectively, substantially higher than those in the LB and PA groups (*p* < 0.05). The additive treatment, exposure period, and their combination substantially impacted the contents of pH value, AA, and NH_3_-N (*p* < 0.05). The LA content was substantially influenced (*p* < 0.05) by both the addition treatment and the exposure duration. The PA content was substantially influenced due to the additive treatment (*p* < 0.05), while it had no substantial influence on the exposure i.e., (aerobic) period (*p* > 0.05). The concentrations of LA and PA in HMCS were not substantially influenced by the combination of additive treatment and exposure period (*p* > 0.05).

**Figure 2:**
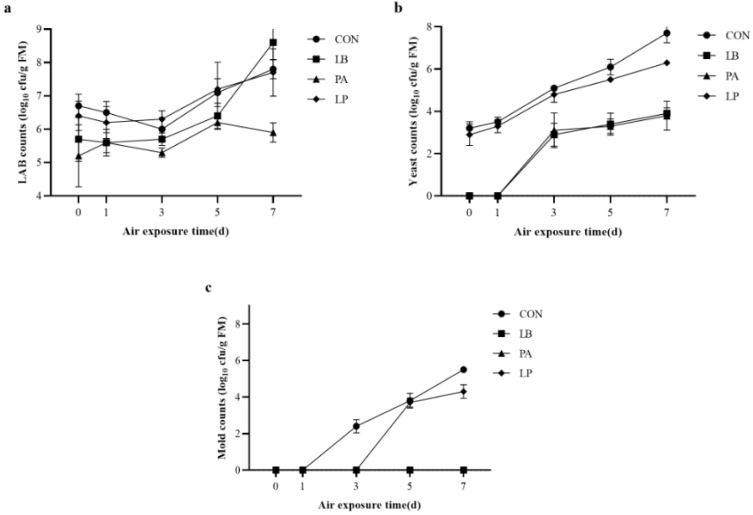
Dynamic changes in lactic acid bacteria count (**a**), yeast count (**b**), and mold count (**c**) of HMCS exposed to air. Treatment: CON, control, no additive; LB, *Lentilactobacillus buchner*; PA, Propionic acid; LP, *Lactiplantibacillus plantarum*.

Figure 2 depicts the variations in the number of microorganisms in HMCS after exposure (aerobic). During the initial five days of exposure (aerobic), LAB were found to be considerably low in the LB and PA test groups in comparison with those in the CON and LP groups (*p* < 0.05). After 7 d of exposure (aerobic), the PA group had the lowest (*p* < 0.05) count of LAB, whereas the LB group had the highest count, substantially greater than the other three groups. After just 1 d of aerobic exposure, the test groups i.e., LB and PA groups started to develop yeast, and after 7 d of aerobic exposure, each test group’s yeast count started to rise. The amount of yeast in the CON and LP groups was noticeably larger in comparison to the other two groups i.e., LB and PA groups after 7 d of aerobic exposure (*p* < 0.05). In the test group, neither the LB group nor the PA group grew any mold throughout the 7 d exposure (aerobic). On the 1 and 3 d, respectively, the CON group and the LP group started to grow mold. Both groups displayed an increasing tendency, peaking on d 7.

## 4. Discussion

HMCS’s chemical makeup was found to be within previously published limits [26,27]. Since the fast build-up of lactic acid leads to the early pH value of silage rapidly dropping, the introduction of acid (lactic acid) bacteria inoculants during the silage process is necessary to ensure effective fermentation [28]. The end pH value following ensiling was mostly utilized in earlier research to assess how LAB inoculants could affect the fermentation quality of silage [29,30]. According to Rezende et al. [31] silage exposed to air has substantial changes in its chemical composition when it comes to oxygen, which is evident in a noticeable rise in temperature and pH.

An efficient antifungal acid that can support aerobic stability is propionic acid. According to Yitbarek et al. [32] the addition of acid (propionic acid) greatly elevated the silage aerobic stability in this study. Acetic acid, which comes from bacteria that ferment heterotrophically, has the potential to modify yeast metabolism and increase silage aerobic stability. This study utilized LAB (LB) for heterotrophic fermentation, which greatly increased high-moisture corn grain silage aerobic stability [33]. Resembling our research findings, previous research [34,35] noted that *L. buchner* raised acetic acid in silage and decreased lactate levels. Nonetheless, it is important to remember that silage’s flavor may be impacted by the overabundance of acetic acid.

According to research by Ranjit and Kung [36], lactobacilli can produce acetic acid even when exposed to oxygen. Acetic acid levels in aerobically farmed silage exhibit an increasing trend, and lactic acid content displays a decreasing trend. The higher pKa of acetic acid in comparison to lactic acid accounts for the pH drop [37]. The findings of our study are supported by Marek Selwet’s research because the changes in the silage’s CP content were not influenced by the additions employed [33].

Comparing LB and PA additives to CON and LP treatments, our findings show a considerable reduction in the average counts of yeast and mold. This is partly due to LB and PA additives increasing the levels of acid, which further has effects on reducing the growth rate of microbes, and the silage’s aerobic stability was also found to be maintained and improved [38]. The amount of yeast and mold in our experiment was substantially affected by the addition of additives, which can quickly lead to the aerobic spoiling of silage feed [39]. The use of LP substantially raises the risk of aerobic instability because it causes the fermentation mode to change toward more acid (lactic acid), which can be utilized as a carbon and energy source by a variety of fungi [39,40,41] and has no inhibitory impact on yeast or mold [40]. Silage feed’s stability can be decreased by introducing homotypic LAB [42,43]. Nonetheless, the LP and CON group aerobic stability in this investigation was comparable. Poor microbial growth may be supported by macromolecules, the contents of which are inversely connected to aerobic stability [44]. This may be the primary cause of the similarities in aerobic stability between the two feed groups. Prior to aerobic exposure, the levels of lactate, soluble carbohydrates, and volatile fatty acids in the LP and CON groups were similar. As per the findings of Cai et al. [45] the LP strain in our study did not hinder yeast growth, which is primarily responsible for aerobic spoiling [44]. Following 5 d aerobic exposure, the quantity of fungi in the LP-inoculated corn and sorghum silage was substantially better than that in the silage of the control group [43].

## 5. Conclusions

The results of our study on the effects of additives on the fermentation process, chemical composition, and aerobic stability of HMCS showed that silage fermentation of freshly harvested corn kernels significantly improved digestibility. In addition, the addition of LB not only significantly improved the quality of HMCS but also significantly improved the aerobic stability of HMCS. Therefore, the use of LB as an additive is recommended in the practical production of HMCS.

## Figures and Tables

**Table 1:** Chemical composition of raw material.

Items	Corn
DM (g kg^−1^ FW)	700.45
aNDF (g kg^−1^ DM)	88.36
ADF (g kg^−1^ DM)	31.83
WSC (g kg^−1^ DM)	51.33
CP (g kg^−1^ DM)	83.06
Starch (g kg^−1^ DM)	71.57
EE (g kg^−1^ DM)	33.15
Ash (g kg^−1^ DM)	7.81
IVDMD (g kg^−1^ DM)	701.56

FW, fresh weight; DM, dry matter; aNDF, neutral detergent fiber; ADF, acid detergent fiber; WSC, water soluble carbohydrate; CP, crude protein; EE, ether extract; IVDMD, in vitro dry matter digestibility.

**Table 2:** Fermentation characteristics of corn grain silage.

Items ^1^	Treatment ^2^				SEM ^3^	*p*-Value
	CON	LB	PA	LP		
pH	3.88	3.84	3.85	3.90	0.023	0.285
LA (g kg^−1^ DM)	5.62 ^c^	8.85 ^a^	6.68 ^b^	8.91 ^a^	0.052	˂0.001
AA (g kg^−1^ DM)	2.12 ^c^	5.46 ^a^	3.18 ^b^	2.14 ^c^	0.026	˂0.001
PA (g kg^−1^ DM)	1.73 ^b^	1.74 ^b^	2.26 ^a^	1.71 ^b^	0.052	˂0.001
NH_3_-N (g kg^−1^ TN)	2.24	2.17	2.14	2.09	0.111	0.14

^a–c^ Means in the same row differed (*p* < 0.05). ^1^ LA, lactic acid; AA, acetic acid; PA, propionic acid. NH_3_-N, ammonia nitrogen. ^2^ CON, control, no additive; LB, *Lentilactobacillus buchner*; PA, Propionic acid; LP, *Lactiplantibacillus plantarum*. ^3^ SEM, standard error of the mean.

**Table 3:** Chemical composition and in vitro dry matter digestibility of high moisture corn grain silage.

Items ^1^	Treatment ^2^				SEM ^3^	*p*-Value
	CON	LB	PA	LP		
DM (g kg^−1^ FW)	663.63 ^b^	678.54 ^a^	676.48 ^a^	679.69 ^a^	0.08	˂0.001
aNDF (g kg^−1^ DM)	70.41 ^a^	63.62 ^c^	66.93 ^b^	63.45 ^c^	0.17	˂0.001
ADF (g kg^−1^ DM)	29.35 ^a^	25.47 ^b^	28.16 ^a^	25.73 ^b^	0.08	˂0.001
Starch (g kg^−1^ DM)	699.93	690.51	681.82	692.55	0.49	0.244
CP (g kg^−1^ DM)	773.14	768.95	774.33	765.39	0.11	0.143
EE (g kg^−1^ DM)	38.42	37.58	37.69	38.17	0.20	0.318
IVDMD (% DM)	81.15	80.77	80.81	81.36	0.03	0.257

^a–c^ Means in the same row differed (*p* < 0.05). ^1^ FW, fresh weight; DM, dry matter; aNDF, neutral detergent fiber; ADF, acid detergent fiber; CP, crude protein; EE, ether extract; IVDMD, in vitro dry matter digestibility. ^2^ CON, control, no additive; LB, *Lentilactobacillus buchner*; PA, Propionic acid; LP, *Lactiplantibacillus plantarum*. ^3^ SEM, standard error of the mean.

**Table 4:** Changes in fermentative characteristics of high moisture corn silage exposed to air.

Items ^1^	Treatment ^2^	Days of Air Exposure (d)					SEM ^3^	*p*-Value ^4^		
		0	1	3	5	7		D	T	D × T
pH	CON	3.88 ^C^	3.89 ^C^	3.91 ^C^	4.97 ^aB^	7.12 ^aA^	0.014	<0.001	<0.001	<0.001
	LB	3.84 ^B^	3.86 ^B^	3.88 ^B^	3.91 ^bB^	4.44 ^cA^				
	PA	3.85 ^B^	3.88 ^B^	3.87 ^B^	3.90 ^bB^	5.31 ^bA^				
	LP	3.90 ^C^	3.89 ^C^	3.87 ^C^	4.86 ^aB^	7.21 ^aA^				
LA (g kg^−1^ DM)	CON	5.62 ^cA^	5.55 ^cA^	5.57 ^cA^	5.48 ^cA^	3.51 ^dB^	1.176	0.007	<0.001	0.178
	LB	8.85 ^a^	8.86 ^a^	8.79 ^a^	8.81 ^a^	8.56 ^a^				
	PA	6.68 ^b^	6.68 ^b^	6.65 ^b^	6.63 ^b^	6.17 ^b^				
	LP	8.91 ^aA^	8.83 ^aA^	8.78 ^aA^	8.24 ^aA^	4.56 ^cB^				
AA (g kg^−1^ DM)	CON	2.12 ^cA^	2.02 ^cA^	1.92 ^cA^	1.15 ^cB^	0.71 ^cB^	0.348	<0.001	<0.001	<0.001
	LB	5.46 ^aA^	5.42 ^aA^	5.27 ^aA^	4.87 ^aB^	4.63 ^aB^				
	PA	3.18 ^bA^	3.07 ^bA^	2.94 ^bA^	2.98 ^bA^	2.66 ^bB^				
	LP	2.14 ^cA^	1.95 ^cA^	1.88 ^cA^	1.24 ^cB^	0.68 ^cC^				
PA (g kg^−1^ DM)	CON	1.73 ^b^	1.74 ^b^	1.69 ^b^	1.72 ^b^	1.69 ^b^	0.321	0.223	<0.001	0.807
	LB	1.74 ^b^	1.74 ^b^	1.72 ^b^	1.73 ^b^	1.68 ^b^				
	PA	2.26 ^a^	2.26 ^a^	2.25 ^a^	2.18 ^a^	2.21 ^a^				
	LP	1.71 ^b^	1.73 ^b^	1.66 ^b^	1.65 ^b^	1.62 ^b^				
NH_3_-N (% TN)	CON	0.23 ^C^	0.22 ^C^	0.37 ^aC^	3.84 ^aB^	12.18 ^aA^	0.119	<0.001	<0.001	<0.001
	LB	0.22 ^C^	0.22 ^C^	0.23 ^bC^	0.41 ^c B^	1.42 ^cA^				
	PA	0.21 ^C^	0.23 ^C^	0.24 ^bC^	1.38 ^bB^	3.76 ^bA^				
	LP	0.21 ^C^	0.21 ^C^	0.46 ^aC^	4.05 ^aB^	13.31 ^aA^				

^A–B, a–c^ Means with different uppercase letters within a row and lowercase letters within a column differ (*p* < 0.05). ^1^ LA, lactic acid; AA, acetic acid; PA, propionic acid. NH_3_-N, ammonia nitrogen. ^2^ CON, control, no additive; LB, *Lentilactobacillus buchner*; PA, Propionic acid; LP, *Lactiplantibacillus plantarum*. ^3^ SEM, standard error of the mean. ^4^ D = effect of days of air exposure; T = effect of additives; D × T = the interaction between days of air exposure and treatment.

## Data Availability

The original contributions presented in this study are included in the article. Further inquiries can be directed to the corresponding author.
